# Fellow-Eye Comparison between Phaco-Microhook Ab-Interno Trabeculotomy and Phaco-iStent Trabecular Micro-Bypass Stent

**DOI:** 10.3390/jcm10102129

**Published:** 2021-05-14

**Authors:** Yuji Takayanagi, Sho Ichioka, Akiko Ishida, Aika Tsutsui, Masaki Tanito

**Affiliations:** Department of Ophthalmology, Shimane University Faculty of Medicine, Izumo 693-8501, Japan; y.takayanagi1008@med.shimane-u.ac.jp (Y.T.); sho-ichi.1002@med.shimane-u.ac.jp (S.I.); ishidaki@med.shimane-u.ac.jp (A.I.); aika0408@med.shimane-u.ac.jp (A.T.)

**Keywords:** microhook ab-interno trabeculotomy, Tanito microhook (TMH), iStent trabecular micro-bypass system, intraocular pressure, minimally invasive glaucoma surgery, cataract surgery, fellow-eye comparison

## Abstract

The aim of this study is to compare the surgical efficacy and safety between microhook ab-interno trabeculotomy (µLOT) and iStent trabecular micro-bypass stent implantation when both were combined with cataract surgery in both eyes of patients. Sixty-four glaucomatous eyes (32 participants; mean age, 75.9 ± 7.6 years; 15 men, 17 women) were included retrospectively. Intraocular pressure (IOP), number of antiglaucoma medications, best-corrected visual acuity (BCVA), anterior chamber flare (ACF) and corneal endothelial cell density (CECD) were evaluated preoperatively, as well as 2, 3, 6, and 12 months postoperatively. Surgical complications and interventions were compared between the procedures. The preoperative IOP and medications with µLOT (18.8 ± 5.7 mmHg and 3.0 ± 1.2, respectively) were higher than with the iStent (15.5 ± 3.4 mmHg and 2.7 ± 1.2, respectively) (*p* = 0.0001 and *p* = 0.0437, respectively). At 12 months, the µLOT values (12.6 ± 2.3 mmHg and 2.3 ± 0.9, respectively) were identical to iStent (12.8 ± 2.5 mmHg and 2.3 ± 0.9, respectively) (*p* = 0.0934 and *p* = 0.3251, respectively). At 12 months, the IOP decreased more with µLOT (6.2 mmHg, 29.5%) than iStent (2.7 mmHg, 15.6%) (*p* = 0.0003). The decrease in medications was greater with µLOT (0.7) than iStent (0.4) (*p* = 0.0437). Survival rate of IOP control ≤15 mmHg and IOP reduction ≥20% was significantly higher after µLOT (40.6% at 12 months) than iStent (18.8%) (*p* = 0.0277). The frequency of layered hyphema was significantly greater with µLOT (8 eyes, 25%) than iStent (0 eyes, 0%) (*p* = 0.0048). The increase in the ACF at 2 weeks postoperatively was significantly greater with µLOT than iStent (*p* = 0.0156), while changes in the BCVA and CECD were identical between groups. The fellow-eye comparison showed that the IOP reduction was greater with µLOT than iStent when combined with cataract surgery.

## 1. Introduction

Glaucoma, which is characterized by loss of retinal ganglion cells, is a leading cause of blindness worldwide [1]. Intraocular pressure (IOP) reduction by medications or surgery remains the mainstay of glaucoma treatment [2]. Conventional filtration surgery has been established as the gold standard in glaucoma surgery; however, it is fraught with complications such as bleb scarring, endothelial cell loss and hypotony [3,4]. Therefore, minimally invasive glaucoma surgery (MIGS) has gained popularity as an attractive surgical option for patients with glaucoma [5,6].

For decades, several less-invasive approaches have been recognized as effective treatments especially for open-angle glaucoma (OAG) [7]. We also previously reported a microhook ab-interno trabeculotomy (µLOT) procedure, a novel and less-invasive approach using a microhook device and its efficacy in reducing IOP [8,9,10,11]. Similarly, iStent (Glaukos, San Clemente, CA, USA) trabecular micro-bypass implantation combined with cataract surgery was also associated with greater IOP lowering potential than cataract surgery alone [12,13,14,15]. Overall, MIGS effectively reduces the IOP and the number of antiglaucoma medications. However, few studies have compared the efficacy and safety between iStent and other ab-interno trabeculotomy such as Kahook dual blade [16,17]. Furthermore, no study compared the efficacy between µLOT and iStent. Therefore, it remains unclear whether the IOP lowering effect and its safety of µLOT is superior to that of iStent.

Here, we investigated the efficacy and complications after µLOT combined with cataract surgery in one eye and iStent implantation combined with cataract surgery in the fellow eyes of patients.

## 2. Methods

### 2.1. Participants

The study adhered to the tenets of the Declaration of Helsinki. The institutional review board (IRB) of Shimane University Hospital reviewed and approved the research. Preoperatively, all participants provided written informed consent for surgery; however, the IRB approval did not require that each patient provide written informed consent for publication. Instead, the study protocol was posted at the study institutions to notify participants about the study. Only anonymous data were used in the statistical analyses. We retrospectively included all participants who fulfilled the following inclusion criteria: subjects who were performed surgeries by one surgeon (MT) at Shimane University Hospital or Matsue Red Cross Hospital from December 2016 to March 2020; subjects who underwent microhook ab-interno trabeculotomy (µLOT) combined with cataract surgery in 1 eye and iStent implantation combined with cataract surgery in the fellow eye within 1 week; subject who with no history of previous intraocular or glaucoma surgeries; and subjects who recorded Goldmann applanation tonometry-measured IOPs and number of antiglaucoma medications preoperatively and at 2 weeks (1–3 weeks) and 3 months (2–4 months), 6 months (5–7 months), 9 months (8–10 months), and 12 months (11–14 months).The surgeon chose μLOT for eyes with severe visual disturbance in almost all cases (in only one case, iStent was implanted for an eye with severe visual field disturbance due to the type of glaucoma). The consecutive 64 eyes of 32 participants (mean age ± standard deviation (SD), 75.9 ± 7.6 years; 15 men, 17 women) subjected to the inclusion criteria were recruited in the study. All participants had complete observation periods. There were no exclusion criteria in this study. The following data were collected by medical chart review: age, sex, laterality, glaucoma types (including primary open-angle glaucoma (POAG), exfoliation glaucoma (EXG) and other types of glaucoma), IOP, number of medications, best-corrected visual acuity (BCVA), anterior chamber flare (ACF) counts using the FM-600 laser flare meter (Kowa, Nagoya, Japan), corneal endothelial cell density (CECD) using the EM-3000 specular microscope (Tomey, Nagoya, Japan), visual field mean deviation (MD) (Central 30-2 Program, Humphrey Visual Field Analyzer, Carl Zeiss Meditec, Dublin, CA, USA), and surgical complications. When the deposition of exfoliation material was seen only in one eye (by slit lamp examination), the subject was defined as unilateral EXG, and the other eye was defined as POAG.

### 2.2. Surgical Procedures

Before μLOT or iStent implantation, phacoemulsification cataract surgery was performed through a 2.2 mm wide clear corneal incision created at the 9 to 10 o’clock position (i.e., temporal incision for the right eye and nasal incision for the left eye). A one-piece soft acrylic intraocular lens was inserted into the capsular bag through the same clear corneal incision. In cases that underwent μLOT, spatula-shaped microhooks (M-2215S, 2215R, and 2215L, Inami, Tokyo, Japan) designed specifically for use during μLOT were used. Viscoelastic material (1% sodium hyaluronate, Provisc, Alcon Japan, Tokyo, Japan, or Opegan Hi, Santen Pharmaceutical, Osaka, Japan) was injected into the anterior chamber (AC) through the clear corneal ports created using a 20-gauge micro-vitreoretinal knife (Mani, Utsunomiya, Japan) at the 2 to 3 and 9 to 10 o’clock positions. A microhook was inserted into the AC through the corneal port, and a Swan-Jacob gonioprism lens (Ocular Instruments, Bellevue, WA, USA) was used to observe the angle opposite to the corneal port. The microhook tip was inserted into Schlemm’s canal and moved circumferentially to incise the inner wall of Schlemm’s canal and trabecular meshwork (TM) greater than 3 clock hours. Using the same procedure, LOT was performed at the opposite angle using a microhook inserted through the other corneal port. In total, the LOT extended more than half of the circumference when the incision was made at both nasal and temporal quadrants. In cases that underwent iStent implantation the first-generation iStent device (GTS100R for right eyes and GTS100L for left eyes, Glaukos Japan, Tokyo, Japan) was implanted into Schlemm’s canal through the TM at the inferonasal quadrant under the observation using a Swan-Jacob gonioprism lens. After μLOT or iStent implantation, the viscoelastic material was aspirated, and the corneal ports were closed by corneal stromal hydration. At the end of surgery, a steroid (2 mg of betamethasone sodium phosphate, Rinderone, Shionogi Pharmaceutical at Shimane University Hospital, and 1.65 mg of dexamethasone sodium phosphate, Decadron, Aspen Japan, Tokyo, in Japan at Matsue Red Cross Hospital) was injected subconjunctivally and 0.3% ofloxacin ointment (Tarivid, Santen Pharmaceutical) was applied. Finally, 1.5% levofloxacin (Nipro, Osaka, Japan) and 0.1% betamethasone (Sanbetason, Santen Pharmaceutical) were applied topically four times daily for 3 to 4 weeks postoperatively in all cases.

### 2.3. Statistical Analysis

The study sample size (*n* = 64, i.e., 32 eyes with each procedure) was calculated to provide 89% power to detect an average difference of postoperative IOP reduction of 3.5 mmHg between eyes (6.2 ± 5.6 mmHg in eyes that underwent µLOT vs. 2.7 ± 3.2 mmHg in eyes implanted with the iStent, with a follow-up correlation of 0.16 between eyes) at 12 months postoperatively. Power calculations were based on a type I error of 0.05 and two-sided test.

To adjust for biases derived from the inclusion of both eyes of a patient, reduction of IOP and the number of antiglaucoma medications were compared using mixed effects regression models in which each patient’s identification number was regarded as a random effect, and the time period and glaucoma surgical procedure were regarded as a fixed effect. For the inter-group comparison at each observation period, the Wilcoxon signed-rank test was used for continuous data and Fisher’s exact probability test for categorical data. The estimated survival probability for qualified IOP control was analyzed using Kaplan–Meier curves. Successful IOP control was assessed by survival curve analysis. For survival curve analysis, the cases were regarded as uncensored when the IOP exceeded 15 mmHg (criterion A) or 12 mmHg (criterion B) after 3 months postoperatively, when the IOP reduction was less than 20% after 3 months postoperatively (both definitions), additional glaucoma surgery at any time (both definitions) or loss of light perception at any time (both definitions). The cases other than the uncensored cases were regarded as censored. Use or unuse of antiglaucoma medication was not considered in the survival curve analysis since most of current cases continued medication postoperatively. Log-rank tests were used to assess the difference in survival rate between surgical groups. All statistical analyses were two-sided and *p* = 0.05 was considered statistically significant. The data are expressed as the means ± SD for continuous variables and in numbers and percentage for categorical variables. For statistical analyses, the decimal BCVA recorded was converted to the logarithm of the minimum angle of resolution. Counting fingers, hand motions, light perception and no light perception were regarded, respectively, as decimal VAs of 0.0025, 0.002, 0.0016 and 0.0013 [18]. All statistical analyses were calculated using the JMP Pro statistical software version 14.2 (SAS Institute, Inc., Cary, NC, USA).

## 3. Results

The demographic data of the participants, including age, sex, laterality, types of glaucoma and MD values measured preoperatively are summarized in Table 1. The glaucoma types included were POAG (65.6%), EXG (23.4%) and other glaucoma types (11.0%). The distribution of glaucoma subtypes did not differ significantly between the two groups; however, they approached significance (*p* = 0.0681), i.e., the eyes that underwent µLOT had more EXG than those implanted with the iStent. The eyes that underwent µLOT also had significantly (*p* < 0.0001) more severe visual field defects.

Table 2 shows the comparison of the IOPs and numbers of antiglaucoma medications preoperatively and postoperatively between µLOT and iStent groups. The mean preoperative IOP and number of antiglaucoma medication in the µLOT group were significantly higher than those in the iStent group (*p* = 0.0001 and *p* = 0.0437, respectively). At 12 months postoperatively, the mean IOP and number of antiglaucoma medication in the µLOT group were identical to those of the iStent group (*p* = 0.0934 and *p* = 0.3251, respectively).

**Figure 1:**
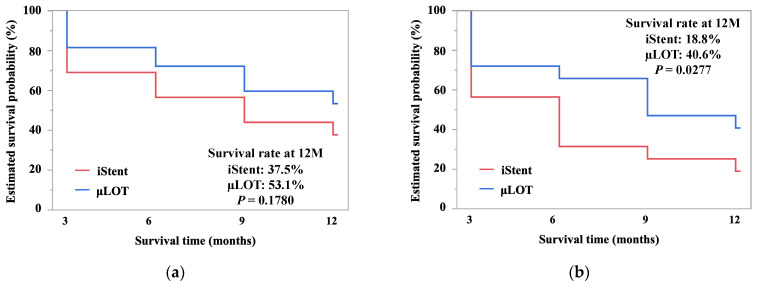
Kaplan–Meier survival curves for successful intraocular pressure (IOP) control in the iStent and microhook ab-interno trabeculotomy (µLOT) groups using two failure criteria, i.e., criterion A (**a**), IOP < 20% reduction from preoperative IOP value and/or >15 mmHg and criterion B (**b**), IOP < 20% reduction from preoperative value and/or >12 mmHg. Patients who did not satisfy the IOP failure criteria and required additional glaucoma surgery and/or who had no light perception were also classified as failures. The log-rank statistics between the two groups were 1.81 for criterion A and 4.85 for criterion B (*p* = 0.1780 and *p* = 0.0277, respectively).

Comparisons of the postoperative reductions in IOP and antiglaucoma medication between the µLOT and iStent groups are shown in Table 3. Mixed effects regression analysis showed that the postoperative reduction of the IOPs and medications differed significantly between the two groups (*p* < 0.0001 for both comparisons). Twelve months postoperatively, decreases in the IOP and the number of medications in the µLOT group were greater than in the iStent group (*p* = 0.0003 and *p* = 0.0437, respectively). Kaplan–Meier survival curves for successful IOP control in both groups are shown in Figure 1. The cumulative survival rates in the iStent and μLOT groups at 12 months were 37.5% and 53.1% for criterion A and 18.8% and 40.6% for criterion B, respectively. The log-rank statistics between the two groups were 1.81 for criterion A and 4.85 for criterion B (*p* = 0.1780 and *p* = 0.0277, respectively).

Table 4 shows the comparison of postoperative complications and interventions between the two groups. The frequency of layered hyphema were significantly higher in the µLOT group than the iStent group (*p* = 0.0048), while the frequency of IOP spikes exceeding 30 mmHg and cystoid macular edema (CME) detected by optical coherence tomography (OCT) were the same (*p* = 1.0000). Additional glaucoma surgery (tube shunt surgery) was required in one eye in the µLOT group.

Comparisons of the preoperative and postoperative BCVAs, ACF and CECD are shown in Table 5. The eyes that underwent µLOT had worse preoperative and early postoperative BCVAs than those implanted with the iStent (*p* = 0.0439). The early postoperative VA in the µLOT group was significantly worse than in the iStent group (*p* = 0.0038); however, this was reversed 3 months postoperatively and the BCVA in the iStent group was significantly worse at 12 months postoperatively (*p* = 0.0072). The early postoperative ACF value in the µLOT group was significantly higher than in the iStent group (*p* = 0.0026). The preoperative CECD in eyes that underwent µLOT was lower than in those implanted with the iStent (*p* = 0.0098)

The postoperative changes in those parameters are shown in Table 6. The postoperative changes in the BCVA did not differ significantly between the two groups. The early postoperative ACF changes in the µLOT group were significantly higher than in the iStent group (*p*= 0.0156). The postoperative changes in the CECD did not differ significantly between the two groups.

## 4. Discussion

The current study compared the efficacy and complications after µLOT and iStent implantation combined with cataract surgery between both eyes of each subject. Overall, the current study identified three major clinical findings. First, µLOT resulted in a greater reduction of IOP and medication postoperatively than implantation of the iStent. Second, the frequency of postoperative layered hyphema was significantly higher in the µLOT group than the iStent group. Third, a higher flare count was found in the early postoperative period in eyes that underwent µLOT compared to eyes implanted with the iStent. To the best of our knowledge, this is the first study to conduct a fellow-eye comparison of IOP lowering between µLOT and the iStent.

The current results show that the eyes which underwent µLOT had a greater reduction in IOP and number of medications postoperatively than those implanted with the iStent. Several studies have reported greater postoperative IOP reductions of excisional goniotomy performed using the Kahook Dual Blade (KDB) (New World Medical, Rancho Cucamonga, CA, USA) compared with the iStent [19,20,21]. Other studies have shown similar or slightly larger IOP reductions with ab-interno goniotomy performed with the Trabectome (NeoMedix, Tustin, CA, USA) than with the iStent [22,23,24,25]. The surgical efficacy of µLOT has been comparable to the KDB [26,27,28] and ab externo LOT [29] in multiple studies, although one study reported less chance of achieving surgical success with µLOT than with the Trabectome [30]. We achieved a 43% IOP decrease from the preoperative value of 25.9 to 14.7 mmHg postoperatively with µLOT alone during the final six month evaluation [31]. When µLOT was combined with cataract surgery, we achieved a 28% decrease, i.e., from 16.4 to 11.8 mmHg postoperatively at the final 9.5 month examination [32]. No study has compared the efficacy of µLOT and iStent. The surgical efficacy of µLOT in the current study was similar to previous reports and indicated that µLOT provided greater reductions in IOP and numbers of medications postoperatively compared with the iStent.

Trabeculotomy lowers the IOP as the result of reduced aqueous flow resistance in the TM [9]. Similarly, the iStent is a trabecular micro-bypass stent that can also effectively lower the IOP safely and less invasively [12,13]. The smaller iStent aperture, with a 120-µm snorkel bore diameter [33], may be more vulnerable to TM reactivity rather than µLOT, during which a wider incision of the inner wall of Schlemm’s canal is created and can sustain aqueous humor drainage [34,35]. This might explain why µLOT produced a greater IOP reduction than iStent in the current study.

We also found that the frequency of postoperative layered hyphema was significantly higher in the µLOT group compared to the iStent group. The frequency of layered hyphema after 1- or 2-quadrant µLOT has been reported to range from 27% to 47% [29,31,32], which is compatible to the current results. One meta-analysis reported that the frequency of hyphema after iStent implantation was 22.2% [13]; however, the frequency of layered hyphema has not been well reported. The potential mechanism for severe hyphema in the µLOT group is that the incision of the inner wall of Schlem’s canal produced by µLOT was extended more than half of the circumference, which was a wider range of incision than that caused by iStent implantation. The current study showed eyes that underwent µLOT developed more severe hyphema, i.e., layered hyphema, compared with those implanted with the iStent.

It is worth noting that the early postoperative flare count was higher in eyes treated with µLOT than with the iStent. We previously reported that the postoperative ACF differed significantly among different glaucoma surgeries including μLOT [36]. More frequent hyphema and more severe inflammation induced by a larger TM incision might explain the higher ACF in eyes that underwent µLOT compared with implantation of the iStent. The correlation among postoperative AC inflammation, frequency of CME and BCVA has been documented previously [37,38,39,40]. Although the frequency of OCT-documented CME did not differ significantly, relatively higher ACF in eyes that underwent µLOT might have resulted in a worse early postoperative VA in the µLOT group compared with the iStent group in the current study. Our results suggest that µLOT might be a more invasive procedure that induces worse AC inflammation than iStent, especially in the early postoperative period. However, this did not affect the final BCVA and our results include the latest evidence on the efficacy and safety of both the µLOT and iStent. Accordingly, monitoring of AC reactions might be important during the early postoperative period after µLOT.

It is also interesting to note that the postoperative changes in the CECD did not differ significantly between the two groups. The early postoperative ACF was higher in the µLOT group, however this inflammation did not affect the postoperative endothelial loss. Theoretically, the inflammation in the anterior chamber may affect the endothelial cell density. However, the duration of postoperative hyphema was relatively short in both procedures. Therefore, the postoperative changes in the CECD did not show statistical significance in this study. Furthermore, the preoperative CECD in eyes that underwent µLOT was lower than in those implanted with the iStent. The higher percentage of EXG in µLOT might explain this difference. Overall, these results may emphasize the safety of µLOT.

The current study had several noteworthy limitations that may affect the generalization of our findings. First, this was a retrospective study and was not controlled or randomized. Second, the senile aged population may limit the generalization of our results. Third, we implanted the iStent in eyes with relatively lower preoperative IOP, more POAG, and mild visual field impairment, which creates a potential selection bias, although the difference in glaucoma types between surgical groups might be cancelled if the unilateral EXG was defined as a bilateral disease. Despite these limitations, our study had many strengths, including the fellow-eye comparison to avoid the confounding effect of patient characteristics, a sufficiently large sample size to detect clinically meaningful differences among all parameters and comprehensive assessments of the patient clinical characteristics including the ACF and CECD.

## 5. Conclusions

In conclusion, the current results showed that the IOP and medication reduction achieved with µLOT was greater than with the iStent when both were combined with cataract surgery, while the achieved IOP levels were identical between both procedures in the fellow-eye comparison. This study highlights the clinical efficacy of µLOT during cataract surgery for reducing IOP and emphasizes that µLOT seems to be an attractive and cost-effective option for patients with glaucoma versus the first generation iStent. The current findings warrant further research to elucidate any differences in the surgical efficacy between different MIGS procedures among µLOT and newer generation device such as iStent inject and iStent inject W.

## Figures and Tables

**Table 1:** Demographic data.

Parameters	μLOT	iStent	*p*
No.	32		
Age (years)			
Mean ± SD	75.9 ± 7.6		
range	59.5, 88.5		
Sex			
Men, *n* (%)	15 (46.9)		
Women, *n* (%)	7 (53.1)		
Laterality			
Left, *n* (%)	14 (43.8)	18 (56.3)	0.4536
Right, *n* (%)	18 (56.3)	14 (43.8)	
Glaucoma types			
POAG, *n* (%)	17 (53.1)	25 (78.1)	0.0681
EXG, *n* (%)	12 (37.5)	3 (9.4)	
Others, *n* (%)	3 (9.4)	4 (12.5)	
MD (dB)			
Mean ± SD	−16.3 ± 8.0	−5.7 ± 6.2	<0.0001 **
range	−30.8, −3.57	−27.2, 1.54	
Severity of visual field defects			
Mild (MD > −6 dB), *n* (%)	3 (10.3)	19 (63.3)	<0.0001 **
Moderate (−12 < MD < −6 dB), *n* (%)	9 (31.0)	7 (23.3)	
Severe (MD < −12 dB), *n* (%)	17 (58.6)	4 (13.3)	

Comparison between the μLOT and iStent groups using the Wilcoxon signed rank test for continuous data and using Fisher’s exact probability test and G test for categorical data. ** *p* < 0.01. μLOT indicates microhook ab-interno trabeculotomy; *n*, number of participants; SD, standard deviation; POAG, primary open-angle glaucoma; EXG, exfoliation glaucoma; MD, mean deviation; dB, decibel.

**Table 2:** IOP and antiglaucoma medications at preoperative and postoperative visits.

Parameters	IOP (mmHg)			Number of Medications (*n*)		
	μLOT	iStent	*p*	μLOT	iStent	*p*
Preoperative value						
Mean ± SD	18.8 ± 5.7	15.5 ± 3.4	0.0001 **	3.0 ± 1.2	2.7 ± 1.2	0.0437 *
Range	12.0, 43.0	13.0, 25.0		1.0, 5.0	1.0, 4.0	
Two weeks postoperatively						
Mean ± SD	15.3 ± 4.9	14.4 ± 3.7	0.4857	2.0 ± 0.9	2.0 ± 0.9	1.0000
Range	7.0, 29.0	8.0, 24.0		1.0, 3.0	1.0, 3.0	
Three months postoperatively						
Mean ± SD	13.1 ± 4.7	12.9 ± 3.3	0.6022	2.2 ± 0.9	2.2 ± 0.9	1.0000
Range	7.0, 33.0	8.0, 22.0		1.0, 3.0	1.0, 3.0	
Six months postoperatively						
Mean ± SD	12.9 ± 3.3	13.3 ± 2.8	0.1848	2.1 ± 0.9	2.2 ± 0.9	0.3251
Range	9.0, 23.0	9.0, 21.0		0.0, 3.0	1.0, 3.0	
Nine months postoperatively						
Mean ± SD	12.8 ± 3.1	13.2 ± 3.2	0.3131	2.3 ± 0.9	2.3 ± 0.9	0.3251
range	6.0, 20.0	6.0, 19.0		1.0, 4.0	1.0, 4.0	
Twelve months postoperatively						
Mean ± SD	12.6 ± 2.3	12.8 ± 2.5	0.0934	2.3 ± 0.9	2.3 ± 0.9	0.3251
Range	7.0, 18.0	8.0, 18.0		1.0, 4.0	1.0, 4.0	

Comparison between the μLOT and iStent groups using the Wilcoxon signed rank test for continuous data. * *p* < 0.05, ** *p* < 0.01. IOP indicates intraocular pressure; μLOT, microhook ab-interno trabeculotomy; *n*, number of participants; SD, standard deviation.

**Table 3:** Postoperative reduction of IOP and antiglaucoma medications.

Parameters	ΔIOP (mmHg)			ΔMedication (*n*)		
	μLOT	iStent	*p*	μLOT	iStent	*p*
Two weeks postoperatively						
Mean ± SD	−3.4 ± 5.1	−1.1 ± 3.9	0.0543	−0.9 ± 1.2	−0.7 ± 1.0	0.0437 *
Range	−20.0, 6.0	−13.0, 7.0		−4.0, 1.0	−3.0, 2.0	
Three months postoperatively						
Mean ± SD	−5.7 ± 6.4	−2.7 ± 4.3	0.0022 **	−0.8 ± 1.1	−0.5 ± 1.0	0.0437 *
Range	−27.0, 7.0	−10.0, 8.0		−4.0, 1.0	−3.0, 2.0	
Six months postoperatively						
Mean ± SD	−5.9 ± 5.5	−2.4 ± 3.9	0.0018 **	−0.9 ± 1.2	−0.5 ± 1.0	0.0437 *
Range	−24.0, 4.0	−12.0, 4.0		−4.0, 1.0	−3.0, 2.0	
Nine months postoperatively						
Mean ± SD	−6.0 ± 6.4	−2.4 ± 3.7	<0.0001 **	−0.7 ± 1.3	−0.4 ± 1.1	0.0437 *
Range	−31.0, 1.0	−11.0, 4.0		−4.0, 2.0	−3.0, 2.0	
Twelve months postoperatively						
Mean ± SD	−6.2 ± 5.6	−2.7 ± 3.2	0.0003 **	−0.7 ± 1.3	−0.4 ± 1.1	0.0437 *
Range	−28.0, 2.0	−12.0, 1.0		−4.0, 2.0	−3.0, 2.0	

Comparison between the μLOT and iStent groups using the Wilcoxon signed rank test for continuous data. * *p* < 0.05, ** *p* < 0.01. ΔIOP indicates reduction in intraocular pressure; ΔMedication, reduction in the number of medications; μLOT, microhook ab-interno trabeculotomy; *n*, number of participants; SD, standard deviation.

**Table 4:** Postoperative complications and interventions.

Parameters	μLOT	iStent	*p*
Layered hyphema, *n* (%)	8 (25.0)	0 (0.0)	0.0048 **
IOP spikes, *n* (%)	2 (6.3)	2 (6.3)	1.0000
Cystoid macular edema	3 (9.4)	4 (12.5)	1.0000
Additional glaucoma surgery, *n* (%)	1 (3.2)	0 (0.0)	1.0000

Comparisons between the μLOT and iStent groups using Fisher’s exact probability test. IOP spikes are defined as IOP greater than 30 mmHg. ** *p* < 0.01. μLOT indicates microhook ab-interno trabeculotomy; *n*, number of participants; IOP, intraocular pressure.

**Table 5:** Preoperative and postoperative ophthalmologic variables.

Parameters	BCVA (LogMAR)			ACF (pc/msec)			CECD (Cells/mm²)		
	μLOT	iStent	*p*	μLOT	iStent	*p*	μLOT	iStent	*p*
Preoperative value									
Mean ± SD	0.32 ± 0.51	0.23 ± 0.51	0.0439 *	11.0 ± 7.9	10.2 ± 6.3	0.3337	2376.3 ± 408.7	2473.5 ± 387.1	0.0098 **
Range	−0.08, 2.70	−0.08, 2.70		3.4, 36.2	4.3, 26.6		785, 2887	1203, 3068	
Two weeks postoperatively									
Mean ± SD	0.237 ± 0.243	0.162 ± 0.486	0.0038 **	46.9 ± 33.4	34.5 ± 29.5	0.0026 **			
Range	−0.079, 0.824	−0.079, 2.699		10.5, 142.9	7.9, 152.2				
Three months postoperatively									
Mean ± SD	0.129 ± 0.154	0.136 ± 0.469	0.0469 **	20.4 ± 11.9	18.1 ± 7.3	0.3217	2176.3 ± 366.9	2256.0, 415.2	0.2269
Range	−0.079, 0.398	−0.079, 2.602		7.2, 55.6	7.1, 32.1		1182, 3109	1284, 2885	
Six months postoperatively									
Mean ± SD	0.069 ± 0.133	0.119 ± 0.473	0.2637	15.3 ± 8.4	14.2 ± 9.4	0.2228	2242.8 ± 330.1	2265.4 ± 405.2	0.3730
Range	−0.079, 0.398	−0.079, 2.602		5.9, 33.9	4.8, 39.0		1541, 2858	1370, 2696	
Nine months postoperatively									
Mean ± SD	0.072 ± 0.133	0.097 ± 0.472	0.0313 *	12.7 ± 6.4	12.3 ± 7.1	0.3634	2169.4 ±399.7	2262.1 ± 414.6	0.0158 *
Range	−0.079, 0.398	−0.079, 2.602		4.8, 31.3	4.8, 36.4		801, 2832	932, 2852	
Twelve months postoperatively									
Mean ± SD	0.071 ± 0.141	0.097 ± 0.474	0.0072 **	12.1 ± 6.4	11.8 ± 7.7	0.2751	2481.1 ± 386.1	2296.2 ± 365.8	0.2694
Range	−0.079, 0.398	−0.079, 2.602		3.0, 28.0	3.0, 33.0		1134, 2807	1093, 2890	

Comparison between the μLOT and iStent groups using the Wilcoxon signed rank test for continuous data (*n* = 32 for BCVA, and *n* = 30 for ACF and CECD). * *p* < 0.05, ** *p* < 0.01. BCVA, best-corrected visual acuity; LogMAR, logarithm of the minimum angle of resolution; ACF, anterior chamber flare; pc, photocount; msec, millisecond; CECD, corneal endothelial cell density; mm², square millimeter; μLOT, microhook ab-interno trabeculotomy; *n*, number of participants; SD, standard deviation.

**Table 6:** Postoperative changes in ophthalmologic variables.

Parameters	ΔBCVA (LogMAR)			ΔACF (pc/msec)			ΔCECD (cells/mm²)		
	μLOT	iStent	*p*	μLOT	iStent	*p*	μLOT	iStent	*p*
Two weeks postoperatively									
Mean ± SD	−0.081 ± 0.522	−0.072 ± 0.216	0.4292	37.1 ± 37.6	25.4 ± 29.7	0.0156 *			
Range	−2.477, 0.669	−0.778, 0.176		0.0, 158.5	0.0, 137.4				
Three months postoperatively									
Mean ± SD	−0.189 ± 0.459	−0.098 ± 0.206	0.6604	8.7 ± 9.5	7.6 ± 6.6	0.9916	−187.6 ± 399.9	−194.8 ± 300.3	0.8963
Range	−2.477, 0.204	−0.824, 0.255		−6.7, 34.3	−8.3, 22.0		−976, 737	−918, 552	
Six months postoperatively									
Mean ± SD	−0.247 ± 0.447	−0.125 ± 0.220	0.1278	4.1 ± 7.0	3.4 ± 6.2	0.6490	−119.7 ± 400.9	−183.6 ± 367.1	0.7200
Range	−2.398, 0.079	−0.903, 0.097		−7.9, 19.2	−8.3, 20.4		−983, 1008	−1116, 521	
Nine months postoperatively									
Mean ± SD	−0.247 ± 0.439	−0.137 ± 0.197	0.1483	2.6 ± 9.8	1.8 ± 5.6	0.9161	−200.1 ± 358.3	−197.2 ± 303.1	0.5001
Range	−2.398, 0.0792	−0.903, 0.079		−9.7, 42.7	−11.7, 13.6		−983, 546	−1169, 408	
Twelve months postoperatively									
Mean ± SD	−0.248 ± 0.436	−0.137 ± 0.195	0.1364	2.6 ± 9.3	1.7 ± 4.9	0.5053	−118.1 ± 349.6	−178.6 ± 260.4	0.1857
Range	−2.398, 0.079	−0.903, 0.079		−12.1, 41.7	−8.2, 14.0		−819, 722	−822, 504	

Comparison between the μLOT and iStent groups using the Wilcoxon signed rank test for continuous data (*n* = 32 for ΔBCVA, and *n* = 30 for ΔACF and ΔCECD). * *p* < 0.05. ΔBCVA indicates postoperative changes of best-corrected visual acuity; LogMAR, logarithm of the minimum angle of resolution; ΔACF, postoperative changes of anterior chamber flare count; pc, photocount; msec, millisecond; ΔCECD, postoperative changes of corneal endothelial cell density; mm^2^, square millimeter; μLOT, microhook ab-interno trabeculotomy; *n*, number of participants; SD, standard deviation.

## Data Availability

Data is fully available upon reasonable request to corresponding author.

## References

[1] Foster A., Resnikoff S. (2005). The impact of Vision 2020 on global blindness. Eye.

[2] Heijl A., Leske M.C., Bengtsson B., Hyman L., Bengtsson B., Hussein M. (2002). Reduction of intraocular pressure and glaucoma progression: Results from the Early Manifest Glaucoma Trial. Arch. Ophthalmol..

[3] O’Connor J., Soon Ang G., Fan Gaskin J.C., Nguyen D.Q., Crowston J.G. (2014). Wound healing modulation in glaucoma filtration surgery—Conventional practices and new perspectives: Antivascular endothelial growth factor and novel agents (Part II). J. Curr. Glaucoma Pract..

[4] Konopińska J., Deniziak M., Saeed E., Bartczak A., Zalewska R., Mariak Z., Rękas M. (2015). Prospective randomized study comparing combined phaco-express and phacotrabeculectomy in open angle glaucoma treatment: 12-Month follow-up. J. Ophthalmol..

[5] Lavia C., Dallorto L., Maule M., Ceccarelli M., Fea A.M. (2017). Minimally-invasive glaucoma surgeries (MIGS) for open angle glaucoma: A systematic review and meta-analysis. PLoS ONE.

[6] Agrawal P., Bradshaw S.E. (2018). Systematic literature review of clinical and economic outcomes of micro-invasive glaucoma surgery (MIGS) in primary open-angle glaucoma. Ophthalmol. Ther..

[7] Saheb H., Ahmed I.I. (2012). Micro-invasive glaucoma surgery: Current perspectives and future directions. Curr. Opin. Ophthalmol..

[8] Tanito M., Matsuzaki Y., Ikeda Y., Fujihara E. (2017). Comparison of surgically induced astigmatism following different glaucoma operations. Clin. Ophthalmol..

[9] Tanito M., Matsuo M. (2019). Ab-interno trabeculotomy-related glaucoma surgeries. Taiwan J. Ophthalmol..

[10] Tanito M., Sugihara K., Tsutsui A., Hara K., Manabe K., Matsuoka Y. (2021). Midterm results of microhook ab interno trabeculotomy in initial 560 eyes with glaucoma. J. Clin. Med..

[11] Tanito M., Tsutsui A., Manabe K., Mochiji M. (2021). Comparison of outflow facility before and after the microhook ab interno trabeculotomy. Eye.

[12] Le J.T., Bicket A.K., Wang L., Li T. (2019). Ab interno trabecular bypass surgery with iStent for open-angle glaucoma. Cochrane Database Syst. Rev..

[13] Popovic M., Campos-Moller X., Saheb H., Ahmed I.I.K. (2018). Efficacy and adverse event profile of the istent and istent inject trabecular micro-bypass for open-angle glaucoma: A meta-analysis. J. Curr. Glaucoma Pract. DVD.

[14] Kozera M., Konopińska J., Mariak Z., Rękas M. (2021). Effectiveness of istent trabecular microbypass system combined with phacoemulsification versus phacoemulsification alone in patients with glaucoma and cataract depending on the initial intraocular pressure. Ophthalmic Res..

[15] Konopińska J., Kozera M., Kraśnicki P., Mariak Z., Rękas M. (2020). The effectiveness of first-generation istent microbypass implantation depends on initial intraocular pressure: 24-Month follow-up-prospective clinical trial. J. Ophthalmol..

[16] Le C., Kazaryan S., Hubbell M., Zurakowski D., Ayyala R.S. (2019). Surgical outcomes of phacoemulsification followed by istent implantation versus goniotomy with the kahook dual blade in patients with mild primary open-angle glaucoma with a minimum of 12-month follow-up. J. Glaucoma.

[17] Iwasaki K., Takamura Y., Orii Y., Arimura S., Inatani M. (2020). Performances of glaucoma operations with Kahook Dual Blade or iStent combined with phacoemulsification in Japanese open angle glaucoma patients. Int. J. Ophthalmol..

[18] Grover S., Fishman G.A., Anderson R.J., Tozatti M.S., Heckenlively J.R., Weleber R.G., Edwards A.O., Brown J. (1999). Visual acuity impairment in patients with retinitis pigmentosa at age 45 years or older. Ophthalmology.

[19] Elmallah M.K., Seibold L.K., Kahook M.Y., Williamson B.K., Singh I.P., Dorairaj S.K. (2019). 12-Month retrospective comparison of kahook dual blade excisional goniotomy with istent trabecular bypass device implantation in glaucomatous eyes at the time of cataract surgery. Adv. Ther..

[20] Lee D., King J., Thomsen S., Hirabayashi M., An J. (2019). Comparison of surgical outcomes between excisional goniotomy using the kahook dual blade and istent trabecular micro-bypass stent in combination with phacoemulsification. Clin. Ophthalmol..

[21] Dorairaj S.K., Kahook M.Y., Williamson B.K., Seibold L.K., Elmallah M.K., Singh I.P. (2018). A multicenter retrospective comparison of goniotomy versus trabecular bypass device implantation in glaucoma patients undergoing cataract extraction. Clin. Ophthalmol..

[22] Al Yousef Y., Strzalkowska A., Hillenkamp J., Rosentreter A., Loewen N.A. (2020). Comparison of a second-generation trabecular bypass (iStent inject) to ab interno trabeculectomy (Trabectome) by exact matching. Graefe’s Arch. Clin. Exp. Ophthalmol..

[23] Gonnermann J., Bertelmann E., Pahlitzsch M., Maier-Wenzel A.-K.B., Torun N., Klamann M.K.J. (2017). Contralateral eye comparison study in MICS & MIGS: Trabectome^®^ vs. iStent inject^®^. Graefe’s Arch. Clin. Exp. Ophthalmol..

[24] Weiner A.J., Weiner Y., Weiner A. (2020). Intraocular pressure after cataract surgery combined with ab interno trabeculectomy versus trabecular micro-bypass stent: An intrasubject same-surgeon comparison. J. Glaucoma.

[25] Esfandiari H., Taubenslag K., Shah P., Goyal S., Weiner A.J., Severson M.L., Weiner A., Grover D.S., Bussel I.I., Loewen N.A. (2019). Two-year data comparison of ab interno trabeculectomy and trabecular bypass stenting using exact matching. J. Cataract Refract. Surg..

[26] Aoki R., Hirooka K., Goda E., Yuasa Y., Okumichi H., Onoe H., Kiuchi Y. (2021). Comparison of surgical outcomes between microhook ab interno trabeculotomy and goniotomy with the kahook dual blade in combination with phacoemulsification: A retrospective, comparative case series. Adv. Ther..

[27] Omoto T., Fujishiro T., Asano-Shimizu K., Sugimoto K., Sakata R., Murata H., Asaoka R., Honjo M., Aihara M. (2020). Comparison of the short-term effectiveness and safety profile of ab interno combined trabeculotomy using 2 types of trabecular hooks. Jpn. J. Ophthalmol..

[28] Omoto T., Fujishiro T., Asano-Shimizu K., Sugimoto K., Sakata R., Murata H., Asaoka R., Honjo M., Aihara M. (2021). Comparison of 12-month surgical outcomes of ab interno trabeculotomy with phacoemulsification between spatula-shaped and dual-blade microhooks. Jpn. J. Ophthalmol..

[29] Mori S., Murai Y., Ueda K., Sakamoto M., Kurimoto T., Yamada-Nakanishi Y., Nakamura M. (2021). Comparison of efficacy and early surgery-related complications between one-quadrant and two-quadrant microhook ab interno trabeculotomy: A propensity score matched study. Acta Ophthalmol..

[30] Tojo N., Otsuka M., Hayashi A. (2021). Comparison of trabectome and microhook surgical outcomes. Int. Ophthalmol..

[31] Tanito M., Sano I., Ikeda Y., Fujihara E. (2017). Short-term results of microhook ab interno trabeculotomy, a novel minimally invasive glaucoma surgery in Japanese eyes: Initial case series. Acta Ophthalmol..

[32] Tanito M., Ikeda Y., Fujihara E. (2017). Effectiveness and safety of combined cataract surgery and microhook ab interno trabeculotomy in Japanese eyes with glaucoma: Report of an initial case series. Jpn. J. Ophthalmol..

[33] Manning D. (2019). Real-world case series of istent or istent inject trabecular micro-bypass stents combined with cataract surgery. Ophthalmol. Ther..

[34] Tanito M. (2021). Delayed-Onset, recurrent hyphema after microhook ab interno trabeculotomy. Case Rep. Ophthalmol..

[35] Tsutsui A., Hamanaka T., Manabe K., Kaidzu S., Kumasaka T., Tanito M. (2021). Histologic findings of trabecular meshwork and schlemm’s canal after microhook ab interno trabeculotomy. J. Glaucoma.

[36] Tanito M., Manabe K., Mochiji M., Takai Y., Matsuoka Y. (2019). Comparison of anterior chamber flare among different glaucoma surgeries. Clin. Ophthalmol..

[37] Ursell P.G., Spalton D.J., Whitcup S.M., Nussenblatt R.B. (1999). Cystoid macular edema after phacoemulsification: Relationship to blood-aqueous barrier damage and visual acuity. J. Cataract Refract. Surg..

[38] Ersoy L., Caramoy A., Ristau T., Kirchhof B., Fauser S. (2013). Aqueous flare is increased in patients with clinically significant cystoid macular oedema after cataract surgery. Br. J. Ophthalmol..

[39] Chu L., Wang B., Xu B., Dong N. (2013). Aqueous cytokines as predictors of macular edema in non-diabetic patients following uncomplicated phacoemulsification cataract surgery. Mol. Vis..

[40] De Maria M., Iannetta D., Cimino L., Coassin M., Fontana L. (2020). Measuring anterior chamber inflammation after cataract surgery: A review of the literature focusing on the correlation with cystoid macular edema. Clin. Ophthalmol..

